# Improved Enzymatic Properties of Chitosanase CsnMY002 from *Bacillus subtilis* via Computational Design

**DOI:** 10.3390/ijms26041588

**Published:** 2025-02-13

**Authors:** Jie Xie, Jingwei Liu, Si Wang, Ganggang Wang

**Affiliations:** 1 Key Laboratory of Environmental and Applied Microbiology, Chengdu Institute of Biology, Chinese Academy of Sciences, Chengdu 610041, China; xiejie@cib.ac.cn (J.X.); liujw@cib.ac.cn (J.L.); wangcai22@mails.ucas.ac.cn (S.W.); 2 Key Laboratory of Environmental Microbiology of Sichuan Province, Chengdu Institute of Biology, Chinese Academy of Sciences, Chengdu 610041, China; 3 University of Chinese Academy of Sciences, Beijing 100049, China

**Keywords:** chitosanase, MD simulations, computational design, thermostability, catalytic activity

## Abstract

Chitooligosaccharides (COSs) are a class of functional carbohydrates with significant application prospects in food and medicine. Chitosanase CsnMY002 from the GH46 family has been used to prepare COS with controlled degrees of polymerization. To enhance the industrial applicability of CsnMY002, molecular dynamics (MD) simulations were applied to investigate the structure–property relationship. Guided by the simulation results, the beneficial mutants were screened through a synergistic strategy using a residue-folding free energy calculation and consensus sequence analysis. Iterative combinations constructed the mutant Mut6 (A49G/K70A/S84A/N89G/D199R/N221G) with significantly improved thermal stability, which had a half-life (*t*_1/2_ value) at 55 °C and 75 °C that was 1.80 and 1.62 times higher than that of the wild type, respectively. A highly active mutant, Mut2, was created, exhibiting a 1.52 times catalytic efficiency of the wild type. An MD simulation analysis of the mutants suggested that the improved enzymatic properties were highly correlated with changes in the dynamic behaviours of the enzyme structure. This study generated more suitable CsnMY002 variants for COS production and provided a comprehensive strategy for the optimization of other industrial enzymes with application potential.

## 1. Introduction

Chitosanases catalyze the hydrolysis of the chitosan to produce well-defined low-molecular-weight chitooligosaccharides (COSs) [1,2,3]. Various research efforts have revealed the medicinal physiological effects of COS, including antibacterial, antioxidant, anti-inflammatory, and anti-tumour activities [4,5,6,7,8]. Therefore, the industrial production of COS by chitosanases has received considerable attention. Based on amino acid sequence similarity, chitosanases can be classified into eight groups, corresponding to glycoside hydrolase (GH) families 2, 3, 5, 7, 8, 46, 75, and 80 (http://www.cazy.org/, accessed on 11 January 2024, Carbohydrate Active Enzyme Database). Among them, the members of the GH46 family have been most extensively characterized [9]. It has been shown that the GH46 chitosanases comprise two domains with a substrate cavity between them, and this cavity contains a large number of negatively charged acidic residues that attract naturally cationic chitosan substrates to bind to the catalytic centre [10]. There are two key catalytic residues (Asp and Glu) on either side of the bound glycosyl chain that function as a general base and general acid to nucleophilically attack and protonate the glycosidic oxygen, respectively [11,12]. Although GH46 family chitosanases share folding and catalytic machinery, the differences in amino acid sequences often result in structural uniqueness of chitosanases from different sources [9]. The understanding of the structure and catalytic mechanism of chitosanase has provided a solid basis for its engineering modification.

With the rapid development of enzymatic COS preparation technologies, higher-performance chitosanases are required to be suitable for challenging industrial environments. Up until now, several strategies have been conducted to improve the properties of chitosanases. Sheng et al. increased the *t*_1/2_ value of an EAG1 mutant from 10.5 min to 69.3 min at 50 °C by introducing stabilizing disulfide bonds [13]. Zhou et al. compared chitosanase CsnTS from *B. subtilis* with thermostable homologs and extended the *t*_1/2_ value of CsnTS at 60 °C from 5.3 min to 55.3 min by residue substitutions [14]. Moreover, a saturation mutagenesis strategy using directed evolution was applied to modify different chitosanases [15,16]. In general, the variable regions of enzyme structure are closely related to catalytic activity and stability [17]. However, studies on the structural dynamics of chitosanases are still lacking. Therefore, uncovering the structure–property relationship is crucial for the industrial chitosanase optimization.

Molecular dynamics simulations (MD) have proven to be powerful tools for investigating the dynamic behaviours of enzyme structures. In particular, using MD simulations can identify highly dynamic regions in the enzyme and potential mutation sites beyond enzyme–substrate interaction regions that can then be modified to improve enzymatic properties. In *Phorcysia thermohydrogeniphila*, an iterative strategy based on MD simulations generated variants A220L and A248S of cytidine kinase, which showed a 7.3-fold and 5.4-fold increase in specific activity compared to the wild type, respectively [18]. In *Streptomyces mobaraenesis*, MD simulation-guided modifications of a transglutaminase resulted in an 84% increase in the half-life at 60 °C and a 21% increase in specific activity [19]. These studies illustrated how MD simulations supplied reliable structural insights. Importantly, the emergence of state-of-the-art computational approaches represented by MD simulations has facilitated the development of synergistic strategies involving a consensus sequence analysis, disulfide bond modification, and folding free energy prediction and iterative combination. This promises to reveal the structure–property relationships of chitosanases and direct the use of comprehensive strategies to obtain mutants suitable for industrial applications.

**Figure 1:**
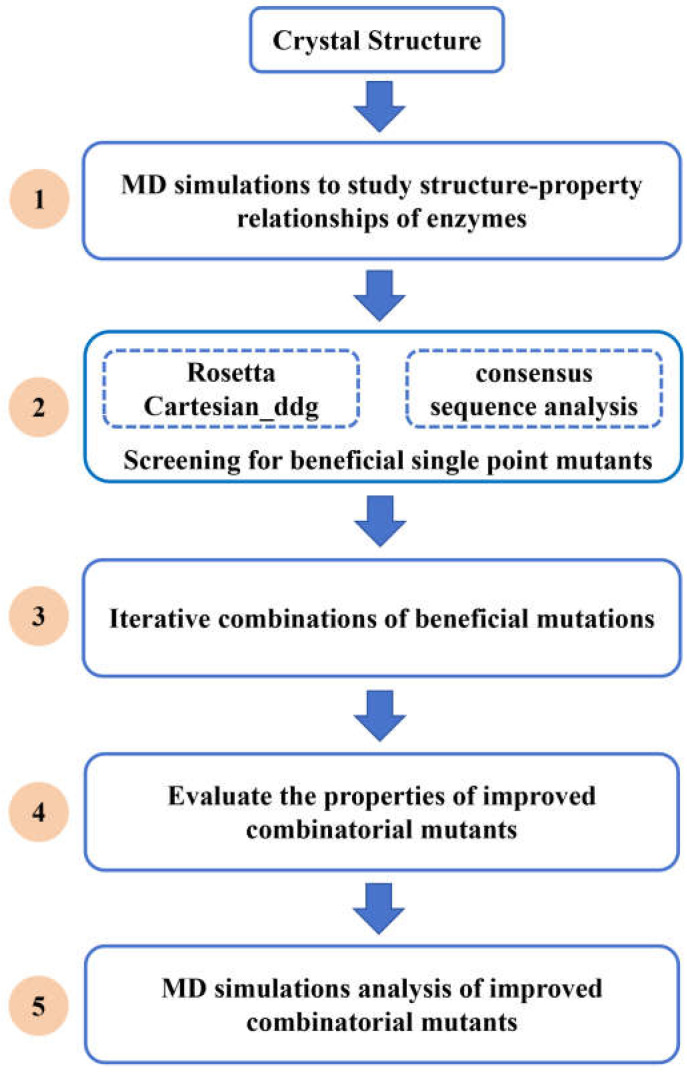
A schematic diagram of the comprehensive strategy to modify the enzymatic properties of chitosanase CsnMY002.

In this report, we used MD simulations to explore the conformational change mechanism of the chitosanase CsnMY002 from *Bacillus subtilis* [20] under thermal stress. To improve the enzymatic properties of CsnMY002, mutation sites were screened through a comprehensive design strategy (Figure 1). Iterative mutagenesis of beneficial single point mutants yielded the mutant Mut2 with increased activity and the mutant Mut6 with significantly enhanced thermal stability. Moreover, MD simulations were performed to investigate the molecular basis for the improved enzymatic properties of the mutants. These results provided new insights into chitosanase engineering for modifying the enzymatic properties, and effective modification strategies can be applied to the engineering of other industrial enzymes.

## 2. Results and Discussion

### 2.1. MD Simulations Reveal Conformational Changes in Chitosanase CsnMY002

**Figure 2:**
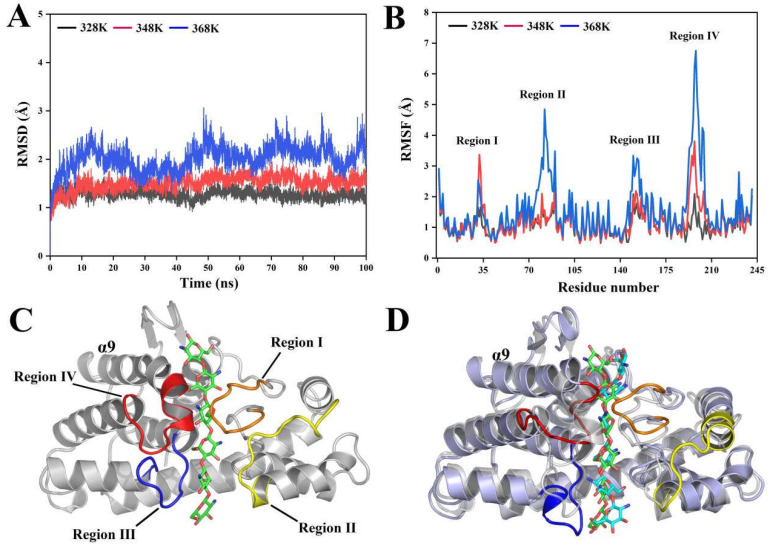
The analysis of the conformational change in CsnMY002 under temperature stress. (**A**) The comparison of the root-mean-square deviation (RMSD) of CsnMY002 at three temperatures. (**B**) The comparison of the root-mean-square fluctuations (RMSFs) of CsnMY002 at three temperatures. (**C**) The crystal structure of CsnMY002. (**D**) The superimposition of the representative CsnMY002 structure at 368 K with the crystal structure. The crystal structure (grey) and the representative MD structure (slate) are shown in cartoon form. Regions I, II, III, and IV in the representative structure are shown in orange, yellow, blue, and red, respectively. The substrates in the crystal and representative MD structure are represented by green and cyan sticks, respectively.

MD simulations were performed to investigate the highly fluctuating conformational regions of CsnMY002 under different temperature stress. The protonated enzyme–substrate complex trajectories were calculated for 100 ns. The analysis of the trajectories showed that the average RMSD values of the Cα atoms in CsnMY002 reached 1.2, 1.5, and 2.0 Å at 328, 348, and 368 K, respectively (Figure 2A). The increase in the backbone RMSD values corresponds to an unstable change in enzyme conformation. As expected, the average radius of gyration (Rg) and solvent-accessible surface area (SASA) values of CsnMY002 gradually increased with elevated temperature, and the average number of internal hydrogen bonds decreased (Figure S1A–C). These results indicated that the fluctuations in the thermosensitive regions of the CsnMY002 structure were exacerbated.

In MD simulations, the RMSF represents the positional fluctuations of residues, with higher RMSF values reflecting greater residue flexibility. Based on the RMSF values, four dynamic regions distributed on either side of the CsnMY002 tunnel-shaped substrate cavity were identified, including regions I (Glu31–Tyr39), II (Ala80–Gly92), III (His147–Phe156), and IV (Met193–Glu203) (Figure 2B,C). The trajectories obtained from MD simulations were then clustered to extract representative conformations. By superimposing the representative conformation at 386 K onto the crystal structure of CsnMY002, the relaxation of the helical structure and the fluctuation of the irregular coiling in these dynamic regions were observed (Figure 2D).

Region I contained two conserved residues in GH46 family chitosanase, the Asp35 at the −1 subsite and the Arg37 at the −2 subsite. These key residues are involved in catalysis and enzyme–substrate-specific interactions [12]. Region III included residues binding the substrate at the −3 and −4 subsites. GH46 family chitosanases are most likely to initially recognize the substrate at negatively numbered subsites and relocate for hydrolysis [11,21]. This implied that the flexibility shown in regions I and III might be essential for catalysis. The functional examination of residues in several GH46 family chitosanases showed that mutants in these regions usually affected the interactions with the substrate and led to a severe loss of enzyme activity [11,12,22].

In contrast, the flexible regions II and IV contained no residues involved in substrate recognition and catalysis, and no development of any possible interactions was observed during the MD simulations. Region II was mainly a flexible coil on the surface of the C-terminal domain, whereas region IV comprised an α9 helical head on the surface of the N-terminal domain and a segment of the loop attached to it (Figure 2D). In CsnMY002, the proximity of the N-terminal and C-terminal structural domains promoted the formation of a tunnel-shaped substrate cavity [20]. Regions II and IV, located on the contact surface of the two structural domains, ensured the stability of the substrate cavity (Figure S2A). Furthermore, regions II and IV also showed a fluctuation correlation with the internal catalytic region (region I) (Figure S2B). This indicated that the flexible regions II and IV might also coordinate the flexibility of region I to support enzyme catalysis. However, the substrate pocket of CsnMY002 experienced a large expansion under MD simulations at 368 K, which caused the exposure of the inner cavity (Table S1 and Figure S3). Meanwhile, the RMSF values of regions II and IV showed marked increases, suggesting that the thermal motion of the surface flexible regions was the primary cause of the structural changes (Figure 2B). It was noticed that the average number of enzyme–substrate hydrogen bonds declined from 20 (348 K) to 15 (368 K) during the 70–100 ns period (Figure S1D). Thus, the extensive relaxation of these surface regions could also lead to an unfavourable catalytic state on the enzyme. Generally, weakening the flexibility of the thermosensitive regions was a key factor in stabilizing the enzyme, while the flexibility of the functional regions was closely related to the enzyme activity [23]. The structure–property relationships revealed by MD simulations indicated that the enhanced stability of the surface regions might be important to improve the thermal resistance of CsnMY002, which enabled the enzyme to build a relatively compact substrate cavity and equilibrate the internal functional regions.

### 2.2. Screening Mutation Sites Using Combinatorial Strategies

MD simulations on the chitosanase CsnMY002 showed that changes in the dynamic behaviours of its surface thermosensitive regions have a correlative effect on the homeostasis of the internal functional regions. To obtain mutants with improved properties, the screening strategies for beneficial mutations guided by structure–property relationships are as follows: (i) Avoid rigid covalent modifications in the surface structure that could lead to severe limitations in the flexibility of the internal functional regions. (ii) Avoid mutating residues in the internal catalytic regions resulting in a severe loss of activity. (iii) Candidate modification sites do not interact with the substrate to avoid affecting the effective recognition of the substrate by the enzyme.

**Figure 3:**
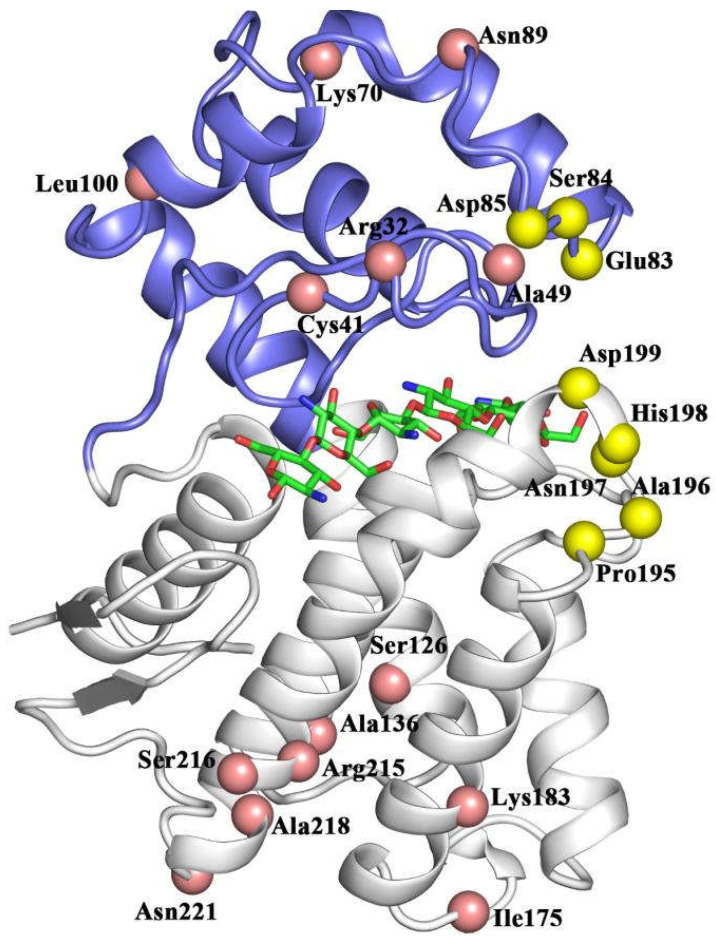
A schematic representation of the candidate sites for mutation in CsnMY002. Sites identified through the consensus sequence analysis are marked by pink spheres, and modification sites targeting flexible regions are marked by yellow spheres. The two structural domains are shown in blue and white. The substrate is shown as a green stick.

The rational design of residues with high RMSF values is a reliable way to modify enzymes [24]. Enhanced enzyme structural stability typically results in reduced folding free energy [19]. Therefore, the eight residues with high fluctuations in regions II and IV (Figure 3) were selected for virtual saturation mutagenesis. Then, the difference in folding free energy (ΔΔG) between the enzyme carrying the point mutation and the wild type was calculated. Based on the ΔΔG values, 10 potentially beneficial mutants were identified, including E83Y, S84A, P195A, A196H, N197Y, N197K, H198L, H198W, D199R, and D199L (Figure S4). Asp85 derivatives were not selected because any mutation would cause higher ΔΔG values (Figure S4).

The evolutionary conserved residues identified in the sequence analysis of homologs are often associated with protein folding and stability [25]. The use of consensus residues to replace variable sites in target proteins has proven to be a successful strategy for improving enzymatic properties [26,27]. The sequence comparison of CsnMY002 with 545 homologs yielded 14 residues with an identity higher than 65% (Table S2 and Figure S5). The majority of these variable sites are distributed on the surface of the two structural domains and have no direct interaction with the substrate (Figure 3). Hence, the 14 variable counterparts in CsnMY002 were to be substituted with consensus residues.

### 2.3. Enzyme Activity- and Thermal Stability-Oriented Mutant Combinations

**Figure 4:**
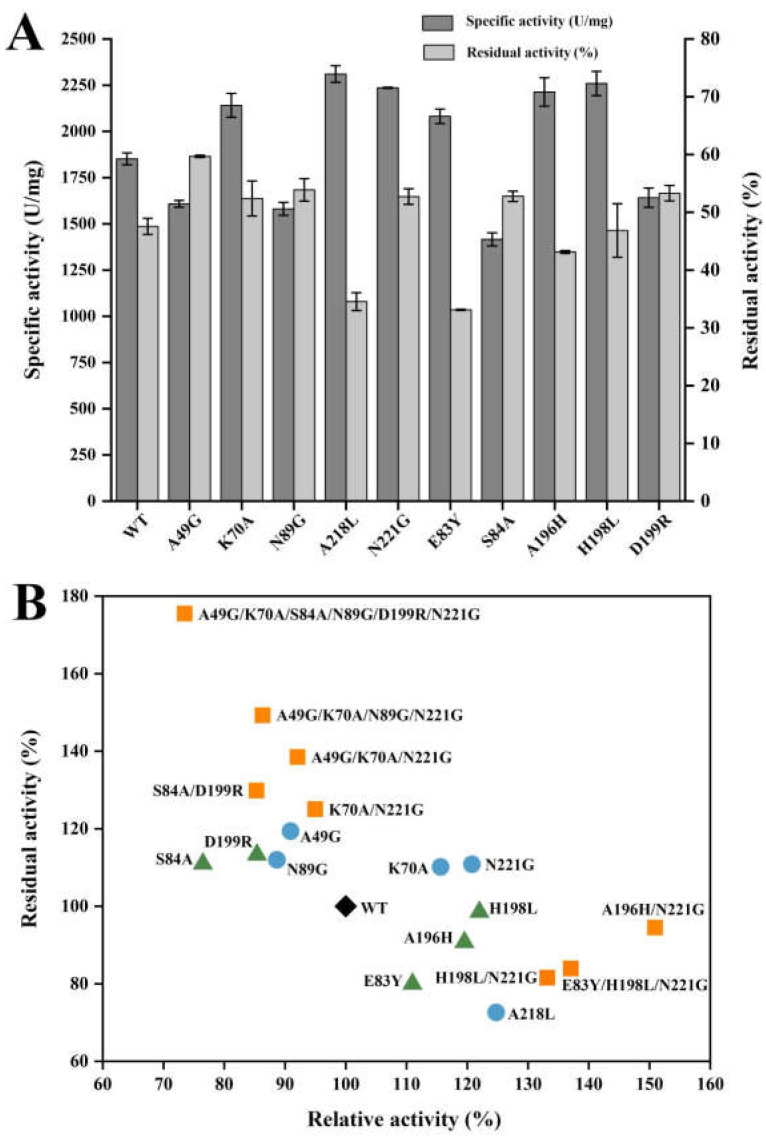
Determination of enzymatic properties of CsnMY002 (wild type, WT) and mutants. (**A**) Initial enzyme activity and residual enzyme activity of wild-type CsnMY002 and favourable mutants. (**B**) Enzymatic properties of combinatorial mutants. Black diamond, wild type. Green triangle, mutants identified through MD simulations. Light blue dots represent mutants identified through consensus sequence analysis. Orange squares represent effective combinatorial mutants.

The 24 screened mutants were successfully expressed and purified (Figure S6). After experimental validation, a total of eight mutants with higher thermal stability were obtained, including A49G, K70A, S84A, N89G, N197K, N197Y, D199R, and N221G, while mutants K70A, E83Y, A196H, H198L, A218L, and N221G showed improved enzyme activity (Table S3). To further improve the enzymatic properties, mutants with a >10% increase in relative activity and remaining > 70% residual activity of the wild type and mutants with a >10% increase in residual activity and remaining > 70% relative activity of the wild type were selected for iterative combination mutagenesis (Figure 4A).

Two mutants with improved thermal stability and relative activity, K70A and N221G, were chosen as templates to incorporate beneficial mutations identified from the consensus sequence analysis. The double mutant K70A/N221G showed 27% higher residual activity and retained about 95% relative activity compared with the wild type (Figure 4B and Table S4). This indicated that the superposition of these two single mutants had a synergistic effect only on thermal stability. Subsequently, A49G and N89G were sequentially introduced into the mutant K70A/N221G. The constructed quadruple mutant (A49G/K70A/N89G/N221G) showed a 49% increase in residual activity and preserved approximately 86% of the relative activity of the wild type (Figure 4B and Table S4). Furthermore, a thermostable double mutant S84A/D199R was obtained by combining two thermal sites located in flexible regions (Figure 4B and Table S4). Because the quadruple mutant (A49G/K70A/N89G/N221G) and the double mutant (S84A/D199R) possessed superior thermal stability and desired catalytic activity, a sextuple mutant of A49G/K70A/S84A/N89G/D199R/N221G (Mut6) was engineered. Mut6 achieved approximately 75% improvement in residual activity (Figure 4B), maintaining over 80% residual activity after incubation at 55 °C for 120 min (Table S4), whereas the wild type lost more than half of enzyme activity under the same conditions (Table S4), indicating that Mut6 has a significant improvement in thermal stability. These results also showed that the combined thermal sites have an additive effect on the thermal stability of CsnMY002. Additionally, single beneficial mutations with improved activity were combined with K70A or N221G to optimize enzyme activity further. Three combinational mutants (A196H/N221G, H198L/N221G, E83Y/H198L/N221G) showed further improvement in enzyme activity (Figure 4B), whereas other combinations had deleterious effects on activity (Table S4). Finally, A196H/N221G (Mut2) was identified as the best combined mutant (Figure 4B and Table S4).

### 2.4. Characterization of Enzymatic Properties

**Figure 5:**
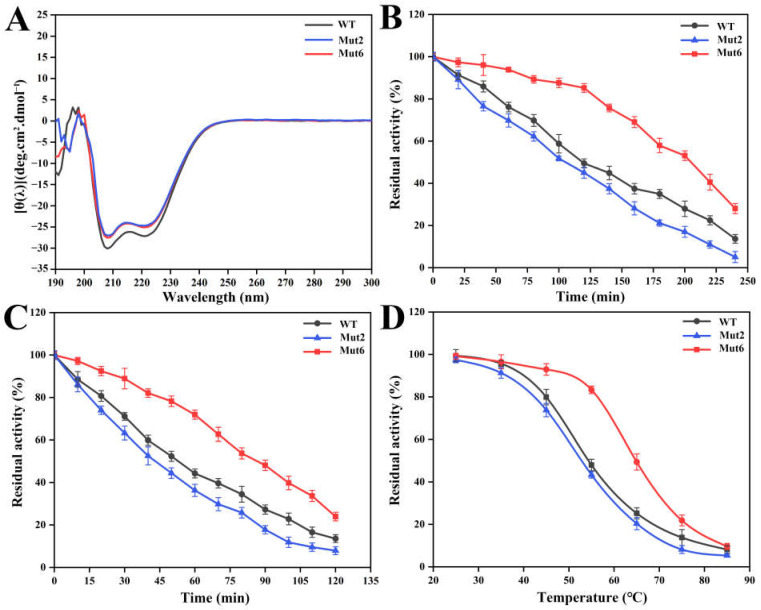
Evaluation of structure and thermal stability of CsnMY002 (wild type, WT) and mutants. (**A**) Analysis of secondary structure features by CD. (**B**) Thermal stability analysis of wild type and mutants at 55 °C. (**C**) Thermal stability analysis of wild type and mutants at 75 °C. (**D**) Determination of half-inactivation temperature (*T*_50_) of wild type and mutants.

Chitosanase CsnMY002 is a typical α helical protein with a rigid structure. The recorded circular dichroism (CD) spectra showed that Mut2 and Mut6 had secondary structure features similar to the wild type, with distinct minima at 208 and 222 nm indicative of the α helical structure (Figure 5A). Thus, the improved enzymatic properties of the mutants did not cause a significant change in overall structure.

The kinetic stability of the wild type and mutant was evaluated by half-life (*t*_1/2_) and half-inactivation (*T*_50_) values. When thermal treatments were carried out at 55 °C and 75 °C for different times, the residual activity of the wild type was observed to decrease faster (Figure 5B,C), with *t*_1/2_ values of 116 min and 53 min, respectively (Table 1). The *t*_1/2_ values of Mut2 remained more than 90% and 80% of the wild type at 55 °C and 75 °C, respectively (Table 1). In contrast, Mut6 had *t*_1/2_ values of 209 min at 55 °C and 86 min at 75 °C, prolonged by approximately 80% and 62% of the wild type, respectively (Table 1). Moreover, the *T*_50_ values of the wild type and Mut2 were comparable, whereas the *T*_50_ value of Mut6 was 10 °C higher than the wild type (Table 1 and Figure 5D).

The *T*_m_ values were measured in the wild type and mutants using differential scanning fluorescence (DSF). The *T*_m_ value represents the thermodynamic stability of the enzyme and is defined as the temperature required for the structural unfolding degree to reach the midpoint. The wild-type CsnMY002 and Mut2 had *T*_m_ values of 57 °C and 56 °C, respectively (Table 1). All of the combined mutants with improved residual activity displayed higher *T*_m_ values (Figure S7), and the optimal mutant Mut6 had a *T*_m_ value of 60 °C (Table 1), indicating enhanced thermodynamic stability.

The optimal reaction conditions of Mut2 and Mut6 were consistent with the wild type (Figure S8). Kinetic constants and catalytic efficiencies were calculated under optimal reaction conditions. The maximum reaction rates (*V*_max_) of the wild type, Mut2, and Mut6 were 3976.17, 5744.35, and 3398.80 µmol·min^−1^·mg^−1^, respectively (Table 1). Mut2 exhibited a higher affinity (lower *K*_m_ value) and substrate conversion rate (*k*_cat_), corresponding to the catalytic efficiency (*k*_cat_/*K*_m_) of 708.67 mL·mg^−1^·s^−1^, which was approximately 52% higher than the wild type (Table 1). The *k*_cat_/*K*_m_ of Mut6 was 328.97 mL·mg^−1^·s^−1^, equivalent to about 71% of the wild type (Table 1).

The GH46 family chitosanases usually have poor thermal stability at relatively high temperatures. It was reported that the chitosanase from *Paenibacillus* sp. 1–18 was almost inactivated after incubation at temperatures exceeding 55 °C for 1 h [28]. The chitosanase originated from *Streptomyces hygroscopicus* R1 had a residual enzyme activity of 23.2% after incubation at 60 °C for 1 h [29]. Through rational enzyme modification on CsnMY002, the activity of the constructed mutant Mut2 was effectively enhanced and retained good thermal stability. In addition, the thermal stability of Mut6 was significantly improved. Compared to the enzyme activity of the best commercially available chitosanases (500 U/mg) [30], Mut6 exhibited a higher specific activity (1361.13 U/mg) (Table S4), which allowed it to be used as a better alternative.

### 2.5. Evaluation of Performance of CsnMY002 Mutants in Hydrolysing Substrates

**Figure 6:**
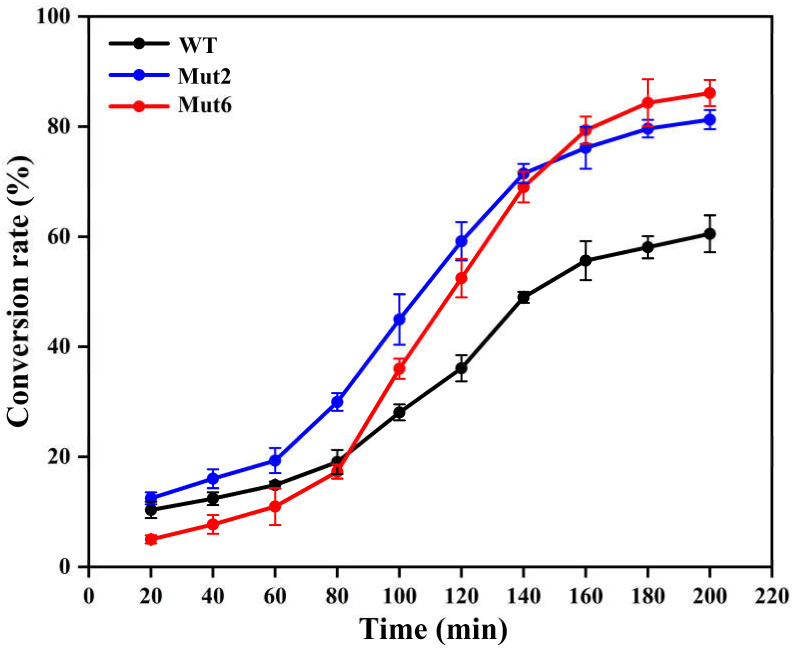
Evaluation of substrate conversion ability by CsnMY002 and mutants.

To further evaluate the application potential of the mutants, Mut2 and Mut6 were employed to hydrolyse high-concentration chitosan substrates in a 100 mL reaction system under optimal reaction conditions. As shown in Figure 6, the wild type reached a conversion rate of 60.5% at 200 min. The Mut2 achieved a substrate conversion rate of 81.3% at the same time. As for Mut6, this mutant exhibited slightly lower substrate conversion in the first 80 min but prevailed over the wild type in the later half, reaching an 86.1% substrate conversion rate at 200 min of hydrolysis (Figure 6). These results further verified that Mut2 and Mut6 converted the substrate more efficiently than the wild type, which are better biocatalysts for COS preparation. The TLC results showed that the cleavage products from Mut2 and Mut6 consisted of DP2 and DP3 (Figure S9), indicating that the modified mutants had a controlled product distribution. The low-molecular-weight COS showed promising biological activities in anti-pathogenic bacteria [31], anti-obesity [32], and antioxidants [33]. The design of Mut2 and Mut6 will facilitate affordable COS being applied into various fields.

### 2.6. Assessing the Stability of Mut2 and Mut6 by MD Simulations

The structures of Mut2 and Mut6 were modelled via AlphaFold2 with CsnMY002 as the template. The best models showed that 95% of the residues were placed in highly plausible regions (Figure S10).

**Figure 7:**
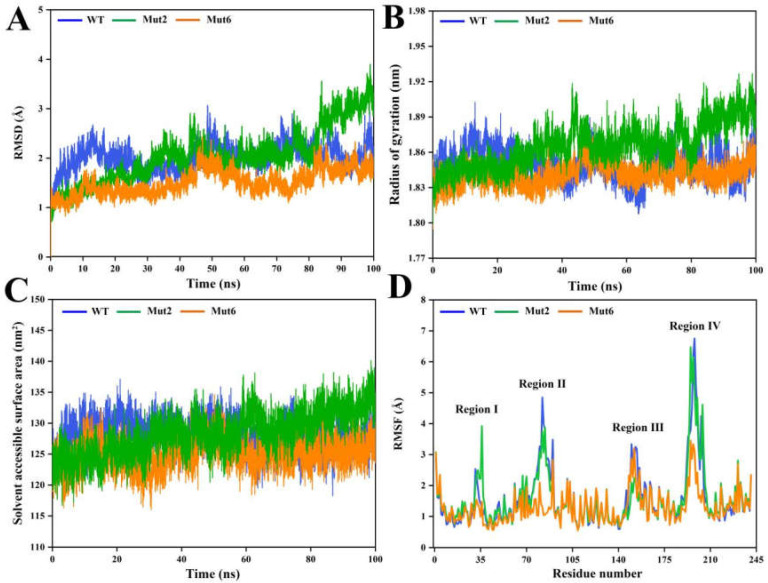
MD simulation analysis of CsnMY002 (wild type, WT) and mutants. (**A**) Comparative RMSD analysis. (**B**) Comparative Rg analysis. (**C**) Comparative SASA analysis. (**D**) Comparative RMSF analysis.

The stability of the mutants Mut2 and Mut6 was investigated by MD simulations at 368 K. During the majority of the 100 ns MD simulations, the RMSD values of Mut6 were an average of 1.5 Å, which is significantly lower than the 2.0 Å RMSD values reported for the wild type (Figure 7A). Therefore, the overall structure of Mut6 exhibited superior rigidity to resist thermal deformation. The RMSD values of Mut2 were lower than the wild type between 0 and 30 ns, reaching a level comparable to the wild type between 30 and 80 ns, and increasing gradually to ~3 Å at the end of the MD simulations (Figure 7A). This result was consistent with experimental data showing that the thermal stability of Mut2 was slightly lower than that of the wild type.

Throughout the MD simulation period, the Rg values of CsnMY002 fluctuated relatively widely over the range of 1.81~1.88 Å (Figure 7B). The Rg values of Mut6 remained constant at ~1.84 Å, whereas the Rg values of Mut2 were greater than the wild type after 30 ns and eventually reached a stable value of ~1.90 Å (Figure 7B). In addition, the average SASA values of Mut6 and the wild type were 125 and 129 nm^2^, respectively (Figure 7C). The SASA values of Mut2 gradually increased over time with an average of 132 nm^2^ during 80 to 100 ns (Figure 7C). The more stable Rg value and lower SASA values further indicated that Mut6 adopted a more compact structure, while the structure of Mut2, which exhibited increased activity, appeared to have relatively susceptible unfolding.

Figure 7D shows the differences in RMSF values. For Mut6, the modified residues might have a coordinated effect in promoting the stabilization of the surface structure, as reflected in the coupled decrease in the RMSF values of regions II and IV (Figure 7D). Moreover, the lower RMSF values in region I were observed (Figure 7D). Region I performs an important catalytic function, and lower flexibility in this region would restrict substrate access and product dissociation. As a result, the improved thermal stability of Mut6 led to a partial sacrifice of catalytic activity. Conversely, Mut2 showed significantly higher RMSF values in region I (Figure 7D); the increase in the flexibility of the internal catalytic regions might contribute to improving enzyme activity. In addition, Mut2 had slightly reduced RMSF values in regions II and III compared with the wild type, but region IV still maintained high RMSF values (Figure 7D).

### 2.7. Structural Analysis of Mut2 and Mut6

**Figure 8:**
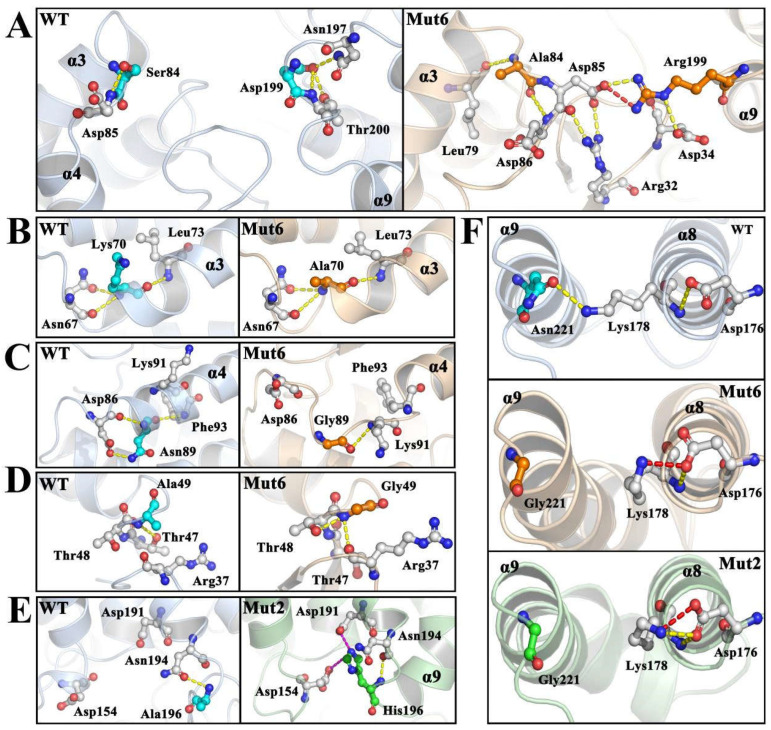
The interaction analysis of CsnMY002 (wild type, WT) and mutants. (**A**) The interaction changes induced by S84A and D199R. (**B**) The interaction changes induced by K70A. (**C**) The interaction changes induced by N89G. (**D**) The interaction changes induced by A49G. (**E**) The interaction changes induced by A196H. (**F**) The interaction changes induced by N221G. The wild-type, Mut2, and Mut6 structures are shown in light blue-, pale green-, and wheat-coloured cartoon representations, respectively. The mutated residues in the wild type, Mut2, and Mut6 are shown as cyan, green, and orange sticks, respectively. The residues interacting with the mutated residues are shown as white sticks. The yellow dashed lines represent hydrogen bonds, the red dashed lines represent salt bridges, and the magenta dashed lines represent electrostatic interactions.

The structures of the wild type, Mut2, and Mut6 after MD simulations were extracted for a further analysis. Mut6 had a more compact structural surface than the wild type in MD simulations (Figure S11A,B), which could account for its significantly different dynamic behaviours. The analysis of the modification sites showed a significant rearrangement of interactions on the Mut6 surface. When Asp199 (region IV) was mutated to Arg199, a new hydrogen bond and salt bridge were formed between Asp85 (region II) and Arg199 (Figure 8A). In addition, Asp85 and Arg199 formed hydrogen bonds with Arg32 and Asp34, respectively (Figure 8A). These interactions could help to enhance the stability of regions II and IV. In region II, the substitution of Ser84 with alanine resulted in the formation of new hydrogen bonds between Ala84 and neighbouring Leu79 and Asp86 (Figure 8A), which facilitated the further stabilization of the loop. Additionally, the presence of Ala84 increased the hydrophobicity and weakened the hydrophilicity of this surface residue. Likewise, Lys70 is located on the surface of the structure; Ala70 has reduced solvent contact and enhanced hydrophobicity (Figure 8B). This altered polarity on the surface residues is considered to be a favourable factor in promoting structural stability [34]. The substitution of Asn89 with Gly89 disrupted hydrogen bonds with Asp86 and Phe93 (Figure 8C). Eliminating these hydrogen bonds facilitated the extension of region II to region IV, favouring enhanced contacts and developed interactions between regions II and IV (Figure 8C), which improved the stability of the enzyme. In contrast, hydrogen bonding of Gly49 with Arg37 and Thr48 stabilized the internal flexible regions (Figure 8D). When Asn221 was replaced by glycine, a hydrogen bond between Asn221 and Lys178 was abolished, and a salt bridge was observed between Lys178 and neighbouring Asp176, which may have improved the rigidity of the local loop on the surface (Figure 8F). Overall, these modifications favoured the establishment of a compact structural surface for Mut6 and promoted the stabilization of the catalytic regions, which could achieve improved thermal stability.

In Mut2, region IV was observed to migrate towards region III, disturbing the contact with region I (Figure S11C). The introduced A196H mutation resulted in electrostatic attraction between His196 and Asp154 and Asp191 (Figure 8E). This promoted the interaction of region IV with region III. Furthermore, the hydrogen bond removal between Asn221 and Lys178 might be beneficial for α9 helical flexibility and affected the dynamic behaviours of region IV (Figure 8F). These changed interactions reduced the contacts of region IV with region I and enhanced catalytic centre flexibility, which could have improved enzyme activity.

Currently, the enzyme modifications have been focused on improving local flexibility or rigidity by eliminating or introducing interactions [35,36,37]. Modifications of the enzymes by disulfide bonds, hydrogen bonds, salt bridges, and hydrophobic interactions at the target regions are all viable representative strategies. Although designing artificial disulfide bonds in the flexible regions could significantly provide the enzyme structure with greater rigidity to enhance thermal stability, it also tends to cause a loss of structural flexibility and significantly reduce catalytic activity [38,39]. The structure–property relationships of enzymes must be considered to direct the proper use of modification strategies and to trade off the thermal stability and activity of the enzyme.

Several studies have been devoted to alleviating the constraints between enzyme catalytic activity and thermal stability. Modifying the flexible terminal structural domain has been reported to achieve a coupled increase in enzyme activity and thermal stability [40,41]. Additionally, one strategy was proposed for flexible regions shifting driven via dynamical network reshaping [17]. By shifting the excessively flexible regions away from the catalytic site, the rigidity of the regions surrounding the catalytic site can be significantly enhanced, thereby improving the stability of the enzyme. The shifted flexible regions could still maintain the overall flexibility of the enzyme structure at a certain level, which would support the conformation change in the enzyme during catalysis and ensure activity. This allowed a trade-off between counteracting mechanisms of enzyme activity and stability.

However, the inspection of the dynamic behaviours of the overall structure of CsnMY002 using MD simulations showed that the thermosensitive regions of CsnMY002 were clustered centrally adjacent to the catalytic pocket, and no obviously flexible regions in the terminal structure were additionally probed for modification. Furthermore, different from other GH46 family chitosanases, the crystal structure revealed that CsnMY002 does not appear to have a more flexible surface structure to open the tunnel-shaped substrate cavity for substrate catalysis via conformation change [20]. Actually, CsnMY002 had a delicate balance between thermal stability and catalytic activity. Despite that regions II and IV located on the surface structure of CsnMY002 exhibited relatively high levels of thermal sensitivity, regions II and IV surrounding the internal catalytic regions not only supported the catalytic homeostasis and structural stability of the enzyme to a certain extent, but also coordinated the internal flexibility required for enzyme catalysis. Shifting the flexible regions II and IV away from the catalytic site favoured the development of significant rigidity in the surface structure to improve thermal stability. It was inevitable that regions II and IV becoming severely rigid would also lock the internal catalytic region (region I), which could result in the activity of CsnMY002 being greatly reduced due to the appropriate flexibility required for catalysis being limited. Therefore, more reliable enzyme modification strategies could be applied based on the elucidation of structure–property relationships.

To date, a variety of tools have been developed to predict potential mutations that could improve the stability and activity of the enzyme [42]. The consensus sequence analysis is one of the most reliable approaches to stabilizing proteins. The prediction of potential substitutions using consensus sequences is typically correct in about half of the results [25]. Furthermore, the prediction tools based on folding free energy changes have been widely developed and applied to optimize industrial enzyme properties, including FireProt 2.0 [43], FoldX 4.0 [44], PROSS [45], FuncLib 2.1.6 [46], and Rosetta Cartesian_ddg version 3.12 [47]. In particular, the Pearson correlation coefficient between the predicted folding free energy changes of over 1000 mutants using Rosetta Cartesian_ddg and experimental measurements reached 0.743 [48]. These high-throughput prediction tools provided reliable accuracy for screening potentially beneficial mutant enzymes. However, it should be noted that the important functional regions of the enzyme could often be mistaken for potential modification sites due to their tendency to show significant flexibility. Therefore, modifications targeting the flexible regions must be based on detailed insights into enzyme–substrate interactions and the catalytic mechanism to avoid severely impairing the function of the flexible regions involved in catalysis.

Here, a comprehensive strategy was performed for improving the enzymatic properties of CsnMY002. The rational design for targeting the flexible regions combined with the consensus sequence analysis yielded 50% beneficial substitutions from the screened single point mutations (Table S3). By gradually accumulating less rigid interactions (e.g., hydrogen bonds, salt bridges, etc.) using an iterative combination strategy, the local structural rigidity of CsnMY002 was adjusted. More than half of combination mutants showed different degrees of improvement in enzyme activity or thermal stability (Table S4). Among them, Mut6 was obtained with significant improvement in thermal stability, exhibiting higher activity than commercially available chitosanases. Mut2 displayed superior catalytic activity and retained thermal stability comparable to the wild type. These two modified mutants could provide suitable candidates for the industrial application of COS enzymatic production.

## 3. Materials and Methods

### 3.1. Strains, Plasmids, and Reagents

*Bacillus subtilis* strain MY002 (CGMCC No. 14841) was maintained in a laboratory. *E. coli* DH5α and *E. coli* BL21 cells were acquired from Novagen (Beijing, China). The pET-28a vector was purchased from Thermo Fisher Scientific (Shanghai, China). Ni-IDA columns were supplied by General Electric Life Sciences and General Electric Healthcare (Pittsburgh, PA, USA). The PrimeStar enzyme was supplied by TaKaRa (Dalian, China). Primers were synthesized by sangon Biotechnology (Chengdu, China). Chitosan (5% acetylation) was purchased from Macklin (Shanghai, China). All other chemicals used were of an analytical grade.

### 3.2. Molecular Dynamics Simulations and Structural Analysis

The structure of CsnMY002 complexed with chitohexose (PDB ID: 7C6D) was used as a starting structure, and the missing catalytic residue Glu19 was added through homology modelling. The chitosanase CsnMY002 efficiently converts chitosan substrates into oligomeric products under weakly acidic conditions (pH 5.5) [20]. Consequently, residues were subjected to pKa calculations using H^++^ (http://newbiophysics.cs.vt.edu, accessed on 13 June 2023), and the protonation states of the residues at pH 5.5 were characterized. The initial coordinates of chitohexose were extracted from the structure of the enzyme–substrate complex and applied to the GAFF force field [49] through hydrogenation, structural modification, and optimization. Finally, the chitohexose used for the simulation represents a positively charged protonated structure.

The modified model of CsnMY002 was subjected to MD simulations using GROMACS (version 2019.3) [50,51] and the AMBER99SB-ILDN force field [52]. Three independent simulations were run at 328, 348, and 368 K. The structure of CsnMY002 was positioned at the centre of a cubic box with periodic boundary conditions, setting the minimum distance between the protein surface and the box to 1.0 nm. The box was solvated with the TIP3P water model, and Na^+^ and Cl^−^ ions were added to neutralize the net charge of the system and provide an ionic strength of 100 mM to the system. Energy minimization was performed on the system using the steepest descent method, with the maximum step size along the gradient direction set to 0.01 nm and the maximum number of steps set to 50,000. To achieve thermodynamic stability under the specified conditions, the temperature of the simulated system was stabilized to a set value through NVT equilibration for 1000 ps and then NPT equilibration for 1000 ps to ensure that the pressure and density of the system were constant. Finally, the work was submitted to the Beijing Super Cloud Computing Center (BSCC) for 100 ns finished MD simulations, with the simulation time step set to 2 fs and traces saved every 100 ps. The mutants Mut2 and Mut6 were modelled using AlphaFold2 [53] with the CsnMY002 as a template, and then MD simulations were performed at 368 K and pH 5.5, with all other parameters held constant.

GROMACS auxiliary programmes were used to analyze the simulated trajectories. The gmx rms, gmx rmsf, gmx gyrat, gmx sasa, and gmx hbond tools were used to calculate the root-mean-square deviations (RMSDs), root-mean-square fluctuations (RMSFs), radius of gyration (Rg), solvent-accessible surface area (SASA), and hydrogen bonding number, respectively. The gmx cluster was used to extract representative conformations of the enzyme under different MD simulation conditions. The visualization of the protein structures was performed with PyMOL 2.5 (https://pymol.org/, accessed on 17 September 2021). Surface area and volume of substrate cavity were calculated using CavityPlus (http://www.pkumdl.cn:8000/cavityplus/, accessed on 8 October 2023). The dynamical cross-correlation analysis was performed by the DynaMut online server (https://biosig.lab.uq.edu.au/dynamut/, accessed on 15 October 2023).

### 3.3. Virtual Saturation Screening Through Rosetta Cartesian_ddg

Virtual mutation screening of residues in highly flexible regions was performed using the Rosetta Cartesian_ddg protocol [48]. An optimal conformation of the wild type was generated through Rosetta *Relax*, and then Cartesian_ddG was used to compare the difference in folding free energy (∆∆G) between the wild type and variants.

### 3.4. Consensus Sequence Analysis of Chitosanase CsnMY002

The amino acid sequence of chitosanase CsnMY002 was obtained from NCBI (http://www.ncbi.nlm.nih.gov, accessed on 21 October 2021), and a sequence identity analysis was performed using Consensus Finder (http://kazlab.umn.edu/, accessed on 26 September 2023). A total of 14 high-frequency residues with >65% identity were selected by comparing 545 homologous sequences. Finally, the results were visualized using WebLogo 3 (https://weblogo.threeplusone.com/, accessed on 26 September 2023).

### 3.5. Heterologous Expression and Purification of Wild Type and Mutants

Chitosanase CsnMY002 and mutants were expressed and purified as described previously [20]. Briefly, *E. coli* BL21 cells were cultured at 37 °C in an LB medium supplemented with 60 μg/mL kanamycin. When the OD_600_ reached 0.6–0.8, isopropyl β-D-1-thiogalactopyranoside (IPTG) was added to a final concentration of 0.2 mM, and cells were further cultured at 16 °C for 18 h. The harvested cells were resuspended in a lysis buffer (50 mM MES, 20 mM imidazole, 500 mM NaCl, pH 6.0) for sonication and fragmentation, and after the removal of the cell pellet by centrifugation (13,000× *g*, 4 °C, 30 min), the supernatant was loaded onto a Ni-IDA affinity column. The target protein was eluted using the elution buffer (50 mM MES, 100 mM imidazole, 500 mM NaCl, pH 6.0). The final protein samples were analyzed by SDS-PAGE [54]. The primers for the mutants are listed in Table S5 and the mutations were introduced into template DNA by the QuikChange mutagenesis method [55]. The mutants were confirmed by DNA sequencing and then expressed and purified according to the procedure described above.

### 3.6. Assay of Chitosanase Activity

A modified 3,5-dinitrosalicylic acid (DNS) method was used to determine chitosanase activity [56]. Briefly, 50 µL of the diluted enzyme suspension was added to 1950 µL of a chitosan solution (50 mM ammonium acetate, pH 5.5) at a concentration of 0.5% and reacted for 10 min in a 55 °C water bath. Subsequently, 500 µL of the hydrolysate was removed immediately and added to 500 µL of the DNS solution to terminate the reaction. The colour was developed by boiling the sample for 5 min. The reaction was cooled to room temperature and centrifuged at high speed to remove insoluble matter, and the total concentration of reducing sugars was determined by measuring the absorbance at 540 nm. In the above conditions, the enzyme activity of one unit of chitosanase was defined as the amount of enzyme required to produce 1 µmol D(+)-glucosamine equivalent reducing sugar per min.

The relative activity of the enzyme was measured in the range of 35~70 °C and the temperature showing the highest activity was defined as the optimal reaction temperature of the enzyme. The relative activity of the enzyme was measured in the range of pH 3.5~6.5 and the pH showing the highest activity was defined as the optimal reaction pH of the enzyme.

Kinetic parameters of the wild type and mutants were determined according to the Lineweaver–Burk method [57] using chitosan as the substrate (1~5 mg/mL) under optimal reaction conditions. All data are derived from experiments performed in triplicate.

### 3.7. Thermal Stability Evaluation of Enzymes

To screen for mutants with improved thermal stability, the initial enzyme activity was regarded as a control, and the residual activity of the wild type and mutants was determined after incubation at 55 °C for 120 min.

The purified enzyme was incubated at different temperatures (25~85 °C) for 120 min and the residual enzyme activity was then determined under optimal reaction conditions. The temperature at which half of the enzyme activity was lost was defined as the half-inactivation temperature (*T*_50_).

The purified enzyme was incubated at specific temperatures (55, 75 °C) for different time intervals and the residual enzyme activity was determined under optimal reaction conditions. The time required for the loss of half the enzyme activity was the half-life (*t*_1/2_).

The melting temperature (*T*_m_) was measured and the stability of the enzyme conformation was analyzed via differential scanning fluorescence (DSF) [58]. A final concentration of 5 mg/mL of chitosanase was mixed with 10× SYPRO dye in 20 µL of the reaction system (100 mM MES, pH 6.0). DSF measurements were conducted in a CFX 96 real-time PCR instrument (Bio-Rad, Hercules, CA, USA) at an excitation wavelength of 490 nm and an emission wavelength of 575 nm. The incubation temperature was gradually increased from 20 to 95 °C at a rate of 1 °C/min. All measurements were performed in triplicate.

### 3.8. Analysis of Protein Secondary Structure by Circular Dichroism (CD)

Secondary structure characteristics of chitosanase and mutants were analyzed using CD spectroscopy (Chirascan V100, London, UK) at 25 °C. Proteins were diluted to 0.25 mg/mL in 50 mM MES buffer (pH 6.0), and spectra were recorded continuously from 180 to 300 nm in three scans, with a 1 mm path length, a 1 nm bandwidth, and a scan rate of 100 nm/min.

### 3.9. Analysis of Hydrolysis Performance of Wild Type and Mutants

The purified enzyme was mixed with the chitosan solution (50 mM sodium acetate, pH 5.5) at a concentration of 25 mg/mL to provide a concentration of 25 µg/mL of the enzyme in the reaction solution. Subsequently, the mixtures were incubated at 55 °C and samples were taken at intervals. The quantity of reducing sugars in the hydrolysate was determined using the DNS method, and the substrate conversion rate was calculated based on the average molecular weight of the hydrolysate.

### 3.10. Analysis of Hydrolysis Products

The thin-layer chromatography (TLC) method was used to analyze the hydrolysis products of the wild type and mutants [20]. Purified chitosanase was added to an ammonium acetate buffer (100 mM, pH 5.5) containing 1% (*w*/*v*) chitosan substrate and mixed, followed by thorough hydrolysis at 55 °C. The enzyme reaction was terminated with boiling water for 5 min and the insoluble matter was removed by centrifugation at 12,000× *g* for 10 min. Then, 1 µL of the hydrolysate was spotted onto silica film and chromatographed using a mixture of ethyl acetate/ethanol/water/ammonium water = 5:5:4:0.5 (*v*/*v*). The silica film was sprayed uniformly with a solution containing 0.1% (*w*/*v*) ninhydrin and incubated at 100 °C for 5 min to develop the colour and detect the product.

## 4. Conclusions

In this study, a comprehensive design strategy based on insights into the enzyme structure–property relationship was performed to improve the enzymatic properties of the chitosanase CsnMY002. Folding free energy calculations targeting residues in the flexible regions and the substitution of consensus sequences were used to adjust the rigidity of local structures. Combinatorial mutants with improved properties were obtained through the iterative optimization of beneficial single mutations. Compared with the wild type, the *T*_m_ and *T*_50_ values of Mut6 were 3 °C and 10 °C higher, respectively. The *t*_1/2_ value of Mut6 was prolonged to 209 min at 55 °C, which was 1.80 times that of the wild type. In addition, Mut2 was obtained with a catalytic efficiency of 708.67 mL·mg^−1^·s^−1^, which was 1.52 times higher than that of the wild type. MD simulations and a structure analysis indicated that reducing the fluctuation of the surface flexible regions to maintain the catalytic regions homeostasis was key to improve thermostability of CsnMY002, and appropriately increasing the internal catalytic regions flexibility might improve the catalytic efficiency. This work provided an excellent biocatalyst with potential commercial value for the industrial production of COS. These results should give referable guidelines for improving the properties of other enzymes.

## Figures and Tables

**Table 1:** Characterization of kinetic parameters and properties of CsnMY002 and mutants.

Enzyme	*V*max (µmol·min^−1^·mg^−1^)	*K*_m_ (mg/L)	*K*_cat_ (s^−1^)	*K_cat_/K_m_* (mL·mg^−1^·s^−1^*)*	*T*_m_ (°C)	*T*_50_ (°C)	*t*_1/2_ (min)	
							55 °C	75 °C
CsnMY002	3976.17 ± 35.81	4.65 ± 0.17	2156.70 ± 19.42	464.72 ± 12.98	57	54	116	53
Mut2	5744.35 ± 137.09	4.39 ± 0.12	3133.47 ± 74.31	708.67 ± 13.23	56	53	105	43
Mut6	3398.80 ± 101.46	4.80 ± 0.25	1577.12 ± 47.08	328.97 ± 14.91	60	64	209	86

## Data Availability

All data, tables, and figures are included in this manuscript or Supplementary Materials.

## Supplementary Materials

The following supporting information can be downloaded at: https://www.mdpi.com/article/10.3390/ijms26041588/s1.
