# Supplementary material for: Improved Enzymatic Properties of Chitosanase CsnMY002 from Bacillus subtilis via Computational Design
Source: Int J Mol Sci. 2025 Feb 13;26(4):1588. doi: 10.3390/ijms26041588 (PMC11855910; doi:10.3390/ijms26041588)
Supplement: Supplementary file 1 [file ijms-26-01588-s001.zip › ijms-3366535-supplementary.pdf]

Supplementary Materials

Improved enzymatic properties of chitosanase CsnMY002 from *Bacillus subtilis* via computational design

Jie Xie[1,2,3], Jingwei Liu[1,2,3], Si Wang[1,2,3], and Ganggang Wang[1,2]*

1 Key Laboratory of Environmental and Applied Microbiology of Chinese Academy of Sciences, Chengdu Institute of Biology, Chinese Academy of Sciences, Chengdu 610041, China

2 Key Laboratory of Environmental Microbiology of Sichuan Province, Chengdu Institute of Biology, Chinese Academy of Sciences, Chengdu 610041, China

3 University of Chinese Academy of Sciences, Beijing 100049, China

*To whom correspondence should be addressed:

Prof. Ganggang Wang

Key Laboratory of Environmental and Applied Microbiology, Chengdu Institute of Biology, Chinese Academy of Sciences, Chengdu, 610041, China

Tel.: +86-28-82890828; Fax: +86-28-82890828; E-mail: wanggg@cib.ac.cn

Table S1. Primer sequences for site-directed mutagenesis.

Mutants	Primers	Sequences (5’→3’)
A49G	A49G-F	GCAGGCTTTACAACGGGTACCGGGGATGCATTG
	A49G-R	CAATGCATCCCCGGTACCCGTTGTAAAGCCTGC
N89G	N89G-F	GCGATGATACAAGCGGTCTCAAGGGATTCGC
	N89G-R	GCGAATCCCTTGAGACCGCTTGTATCATCGC
N221G	N221G-F	CTATCGCCAAGGAGGGCAACTATAATCTAAAC
	N221G-R	GTTTAGATTATAGTTGCCCTCCTTGGCGATAG
A136G	A136G-F	CTAAAAACAGCATTGGGTAGAGCTGTGATGTAC
	A136G-R	GTACATCACAGCTCTACCCAATGCTGTTTTTAG
R215Q	R215Q-F	CGTGTGGACGTGCTTCAGTCTATCGCCAAGGAG
	R215Q-R	CTCCTTGGCGATAGACTGAAGCACGTCCACACG
R32D	R32D-F	GGATATGTAGAGGACTTGGATGATGGGCG
	R32D-R	CGCCCATCATCCAAGTCCTCTACATATCC
K70A	K70A-F	CCGAATAACAAACTGGCAAAGTATCTGCCTG
	K70A-R	CAGGCAGATACTTTGCCAGTTTGTTATTCGG
S126A	S126A-F	CCTGCCATGAAACGAGCGGATAATGCCGGAC
	S126A-R	GTCCGGCATTATCCGCTCGTTTCATGGCAGG
C41A	C41A-F	GGCGAGGCTATACAGCCGGACGGGCAGGCTTTAC
	C41A-R	GTAAAGCCTGCCCGTCCGGCTGTATAGCCTCGCC
A218L	A218L-F	GTGCTTCGCTCTATCCTCAAGGAGAACAAC
	A218L-R	GTTGTTCTCCTTGAGGATAGAGCGAAGCAC
L100A	L100A-F	CTGCCTGGAAGTCGGCTGCAAATGATAAGG
	L100A-R	CCTTATCATTTGCAGCCGACTTCCAGGCAG
K183A	K183A-F	GAAGAAGTGGTTGAATGCATTCTTGGACGTACGC
	K183A-R	GCGTACGTCCAAGAATGCATTCAACCACTTCTTC
S216R	S216R-F	GTGGACGTGCTTCGCCGTATCGCCAAGGAGAAC
	S216R-R	GTTCTCCTTGGCGATACGGCGAAGCACGTCCAC
I175G	I175G-F	CACCTAAAGACGGAGGAGACGAGAAGAAGTG
	I175G-R	CACTTCTTCTCGTCTCCTCCGTCTTTAGGTG
E83Y	E83Y-F	CGTCTGGCCAAGGAATACAGCGATGATACAAGC
	E83Y-R	GCTTGTATCATCGCTGTATTCCTTGGCCAGACG
S84A	S84A-F	CTGGCCAAGGAAGAAGCCGATGATACAAGCAATC
	S84A-R	GATTGCTTGTATCATCGGCTTCTTCCTTGGCCAG

(Continue) Table S1. Primer Sequences for Site Directed Mutagenesis

Mutants	Primers	Sequences (5’→3’)
P195A	P195A-F	GACGATCTGATGAATGCGGCCAATCATGACACC
	P195A-R	GGTGTCATGATTGGCCGCATTCATCAGATCGTC
A196H	A196H-F	GATCTGATGAATCCGCACAATCATGACACCCGTG
	A196H-R	CACGGGTGTCATGATTGTGCGGATTCATCAGATC
N197K	N197K-F	CTGATGAATCCGGCCAAGCATGACACCCGTGAC
	N197K-R	GTCACGGGTGTCATGCTTGGCCGGATTCATCAG
N197Y	N197Y-F	CTGATGAATCCGGCCTATCATGACACCCGTGAC
	N197Y-R	GTCACGGGTGTCATGATAGGCCGGATTCATCAG
H198L	H198L-F	GATGAATCCGGCCAATCTTGACACCCGTGACGAATG
	H198L-R	CATTCGTCACGGGTGTCAAGATTGGCCGGATTCATC
H198W	H198W-F	GATGAATCCGGCCAATTGGGACACCCGTGACGAATG
	H198W-R	CATTCGTCACGGGTGTCCCAATTGGCCGGATTCATC
D199L	D199L-F	GAATCCGGCCAATCATCTCACCCGTGACGAATGG
	D199L-R	CCATTCGTCACGGGTGAGATGATTGGCCGGATTC
D199R	D199R-F	GAATCCGGCCAATCATCGCACCCGTGACGAATGG
	D199R-R	CCATTCGTCACGGGTGCGATGATTGGCCGGATTC

Note: Mutated codons are highlighted in red and underlined.

Table S2. Statistics on the dimensions of the substrate binding pocket.

CsnMY002	Surface Area (Å^2)	Volume (Å^3)	Box Size (Å)
Crystal structure	986.5	1418.12	18.0×20.0×25.5
Representative structure at 328K	1210.75	1888.62	26.5×21.0×20.0
Representative structure at 348K	1301.00	1894.12	17.5×22.0×26.0
Representative structure at 368K	1350.75	2268.62	25.0×20.0×28.0

Note: The box size represents the length, width and height of the substrate cavity.

Table S3. Candidate sites screened by sequence identity analysis.

Target of mutations	Identity
Change A 49 to G	80% of similar proteins have G, only 11% have A
Change N 89 to G	77% of similar proteins have G, only 3% have N
Change N 221 to G	77% of similar proteins have G, only 5% have N
Change A 136 to G	77% of similar proteins have G, only 8% have A)
Change R 215 to Q	73% of similar proteins have Q, only 9% have R
Change R 32 to D	73% of similar proteins have D, only 3% have R
Change K 70 to A	72% of similar proteins have A, only 4% have K
Change S 126 to A	72% of similar proteins have A, only 3% have S
Change C 41 to A	70% of similar proteins have A, only 7% have C
Change A 218 to L	69% of similar proteins have L, only 9% have A
Change L 100 to A	69% of similar proteins have A, only 5% have L
Change K 183 to A	66% of similar proteins have A, only 9% have K
Change S 216 to R	66% of similar proteins have R, only 5% have S
Change I 175 to G	66% of similar proteins have G, only 7% have I

Note: Sites with a sequence identity exceeding 65% were selected for mutation.

Table S4. Enzyme activity and thermal stability of wild-type and mutants.

Enzyme	Specific activity (U/mg)	Residual enzyme activity (U/mg)	Residual enzyme activity (%)
wild-type	1852.08±32.34	881.09±12.25	47.57
R32D	1047.91±15.30	471.10±0.33	44.96
C41A	1130.14±11.53	467.21±7.62	41.34
A49G	1608.90±18.50	960.50±1.92	59.70
K70A	2141.19±64.45	1122.12±34.00	52.41
N89G	1581.31±34.95	852.25±16.62	53.90
L100A	312.82±3.75	121.82±3.22	38.94
S126A	1374.61±31.75	574.29±25.79	41.78
A136G	1660.20±71.89	692.82±0.69	41.73
I175G	1304.79±24.27	620.11±6.57	47.53
K183A	1710.21±57.12	746.64±32.25	43.66
R215Q	1823.19±24.07	786.51±9.83	43.14
S216R	712.68±13.61	285.54±6.54	40.07
A218L	2310.66±44.83	798.38±12.37	34.55
N221G	2236.57±2.91	1179.24±15.92	52.73
E83Y	2081.91±38.91	698.83±0.69	33.13
S84A	1416.47±35.27	747.58±6.73	52.78
P195A	1758.36±40.62	618.26±2.41	35.16
A196H	2213.79±77.26	955.25±2.29	43.15
N197K	612.85±0.61	324.79±0.39	53.00
N197Y	928.87±12.08	491.24±18.67	52.89
H198L	2259.72±65.76	1058.89±49.03	46.86
H198W	1514.82±4.70	695.85±11.20	45.94
D199L	1811.33±38.40	493.41±1.18	27.24
D199R	1642.05±52.22	875.46±11.82	53.32

Note: All data were obtained from the average of three independent measurements.

Table S5. Enzyme activity and thermal stability of wild-type and combined mutants.

Enzyme	Specific activity (U/mg)	Residual enzyme activity (U/mg)	Residual enzyme activity (%)
wild-type	1852.08±32.34	881.09±12.25	47.57
K70A/N221G	1,759.00±30.45	1,046.33±29.25	59.48
K70A/N221G/A49G	1,705.19±87.80	1,121.51± 12.00	65.77
K70A/N221G/A49G/N89G	1,598.95±56.72	1,135.37±46.77	71.01
S84R/D199R	1580.19±34.49	976.20±13.79	61.78
K70A/N221G/A49G/S84A/N89G/D199R	1361.13±43.23	1,136.32±14.99	83.48
A196H/N221G	2794.97±89.77	1256.34±33.78	44.95
H198L/N221G	2466.80±171.96	957.29±30.97	38.81
E83Y/A196H/N221G	2085.71±106.34	688.09±24.99	32.99
E83Y/H198L/N221G	2538.25±61.48	1013.70±26.93	39.94
A218L/A196H/N221G	2291.93±65.99	675.67±7.01	29.48
A218L/E83Y/H198L/N221G	2637.67±49.79	511.04±7.95	19.37
K70A/A196H	1,595.01±88.07	649.30±35.28	40.71
K70A/H198L	2,125.23±92.65	883.62±42.68	41.58
A196H/H198L	1,119.80±35.94	416.62±5.62	37.21

Note: All data were obtained from the average of three independent measurements.

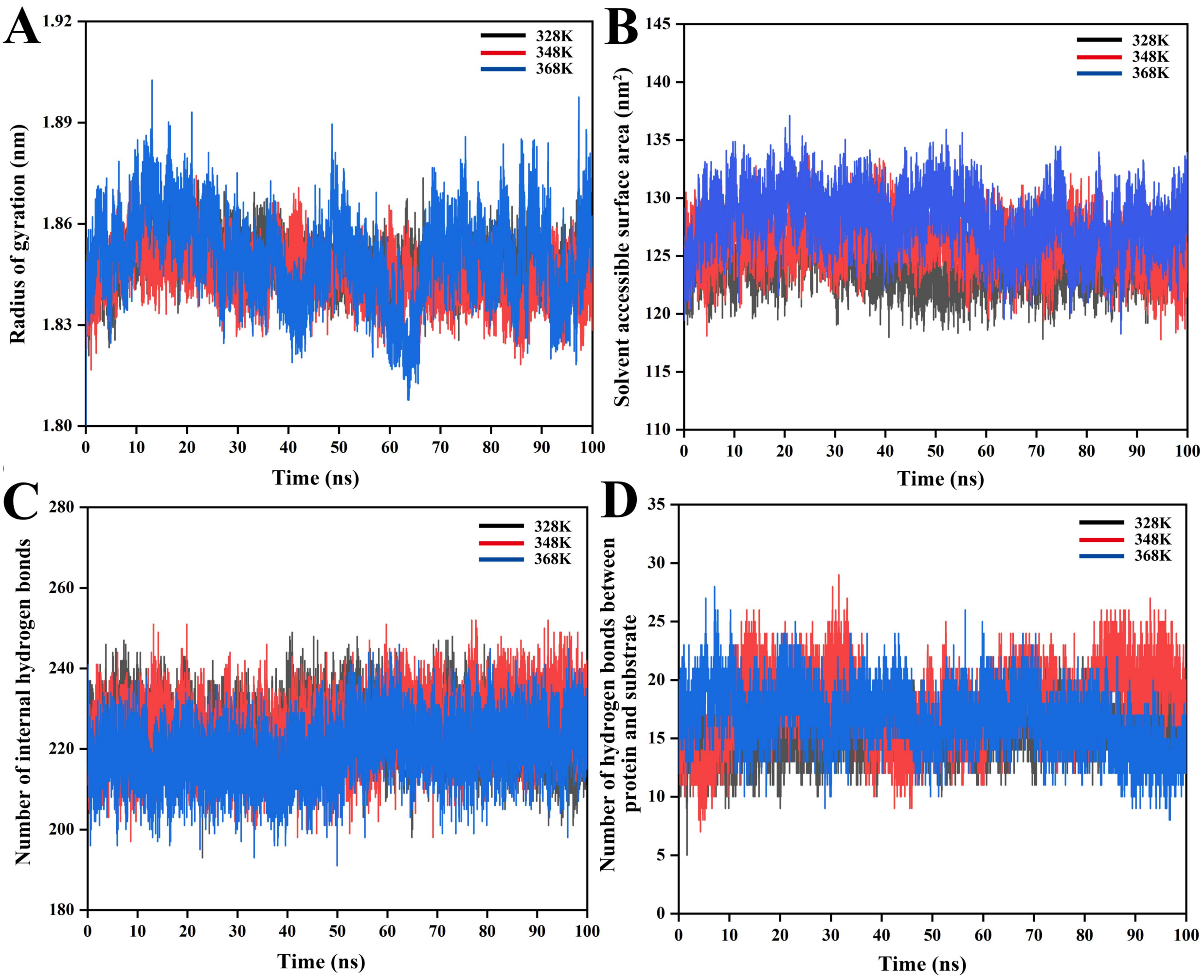

Figure S1. Analysis of the stability of CsnMY002 at different temperatures. (A) Radius of gyration (Rg). (B) Solvent accessible surface area (SASA). (C) Number of internal hydrogen bonds. (D) Number of hydrogen bonds between protein and substrate.

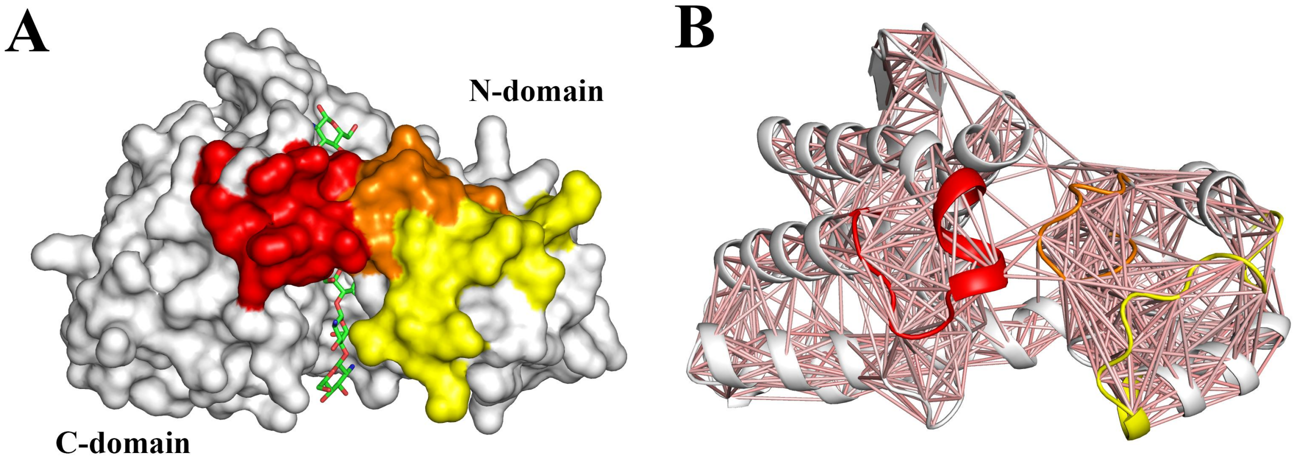

Figure S2. The dynamic correlation analysis between thermosensitive regions (regions II and IV) and catalytic region (region I). (A) The surface structure of CsnMY002. (B) The dynamical cross-correlations at the 3D structure level. The regions I, II and IV are shown in orange, yellow and red, respectively. The substrates are represented by green sticks. The regions with dynamical correlations are linked by pink line segments.

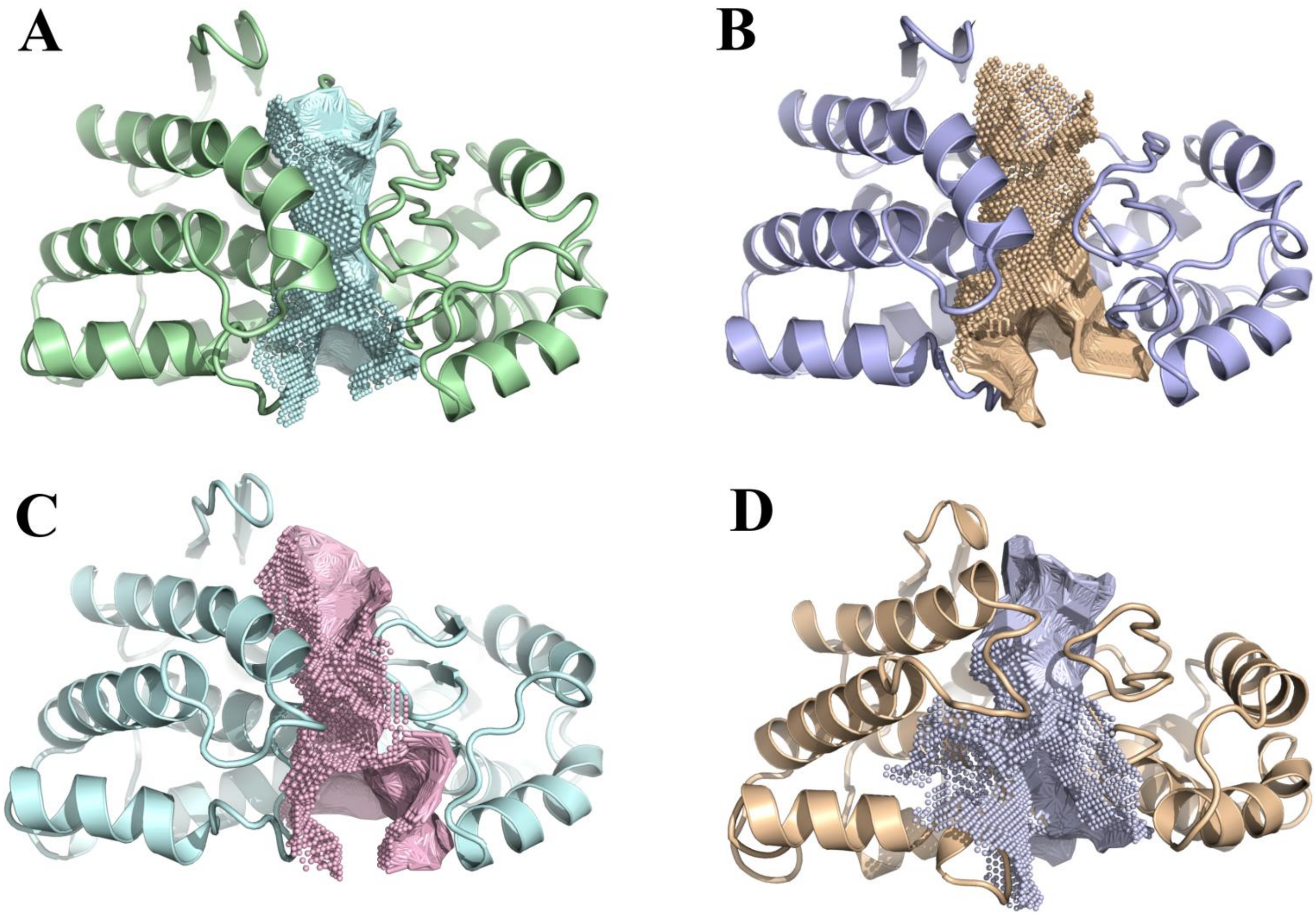

Figure S3. The three-dimensional structure of the substrate binding pocket. (A) Crystal structure of CsnMY002. (B) Representative structure at 328K. (C) Representative structure at 348K. (D) Representative structure at 368K. The crystal structure and the representative structures are shown in cartoon form. The substrate binding pockets are shown in surface form.

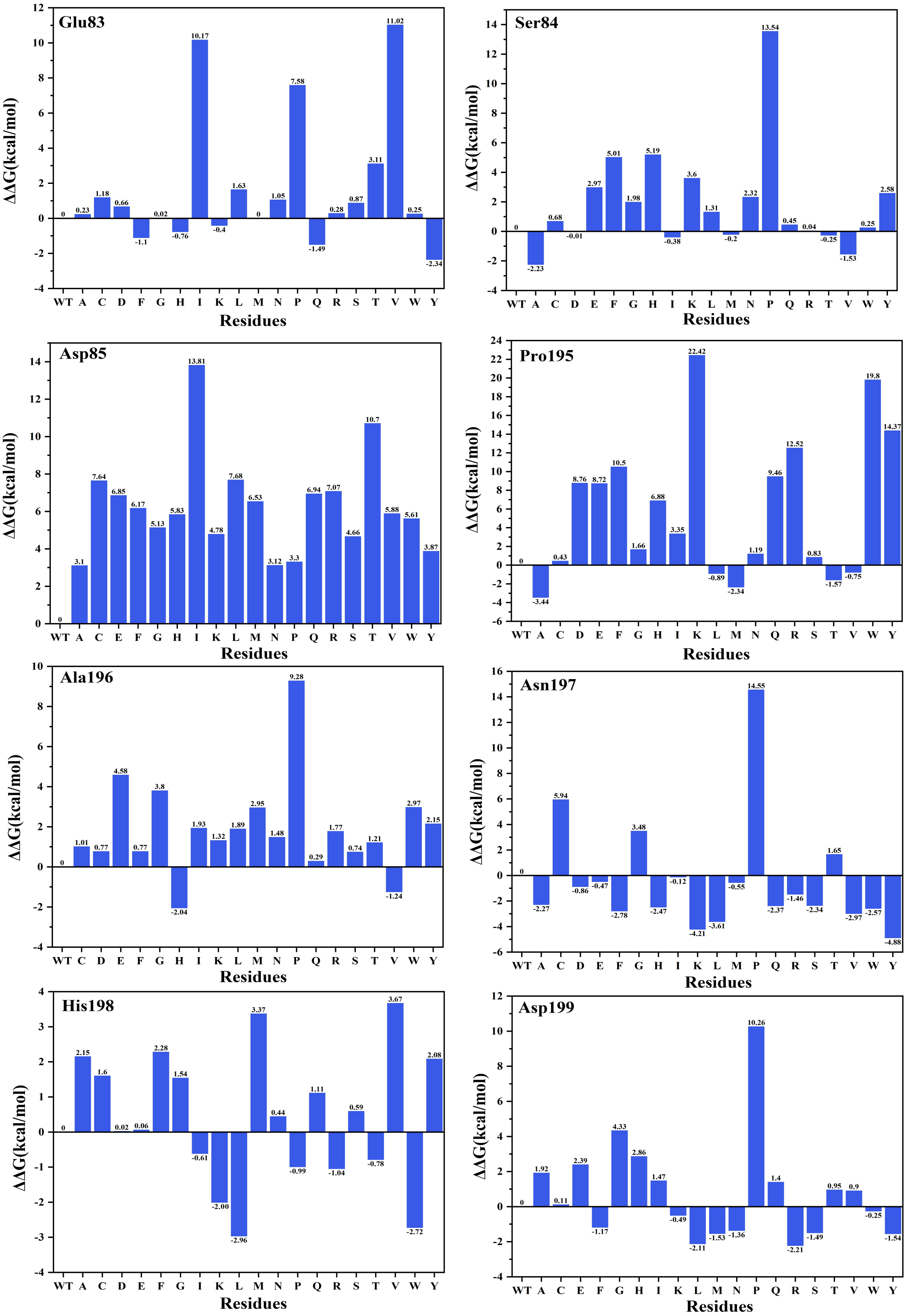

Figure S4. Predicting changes in folding free energy for saturating mutants with high flexible residues using Rosetta Cartesian_ddg.

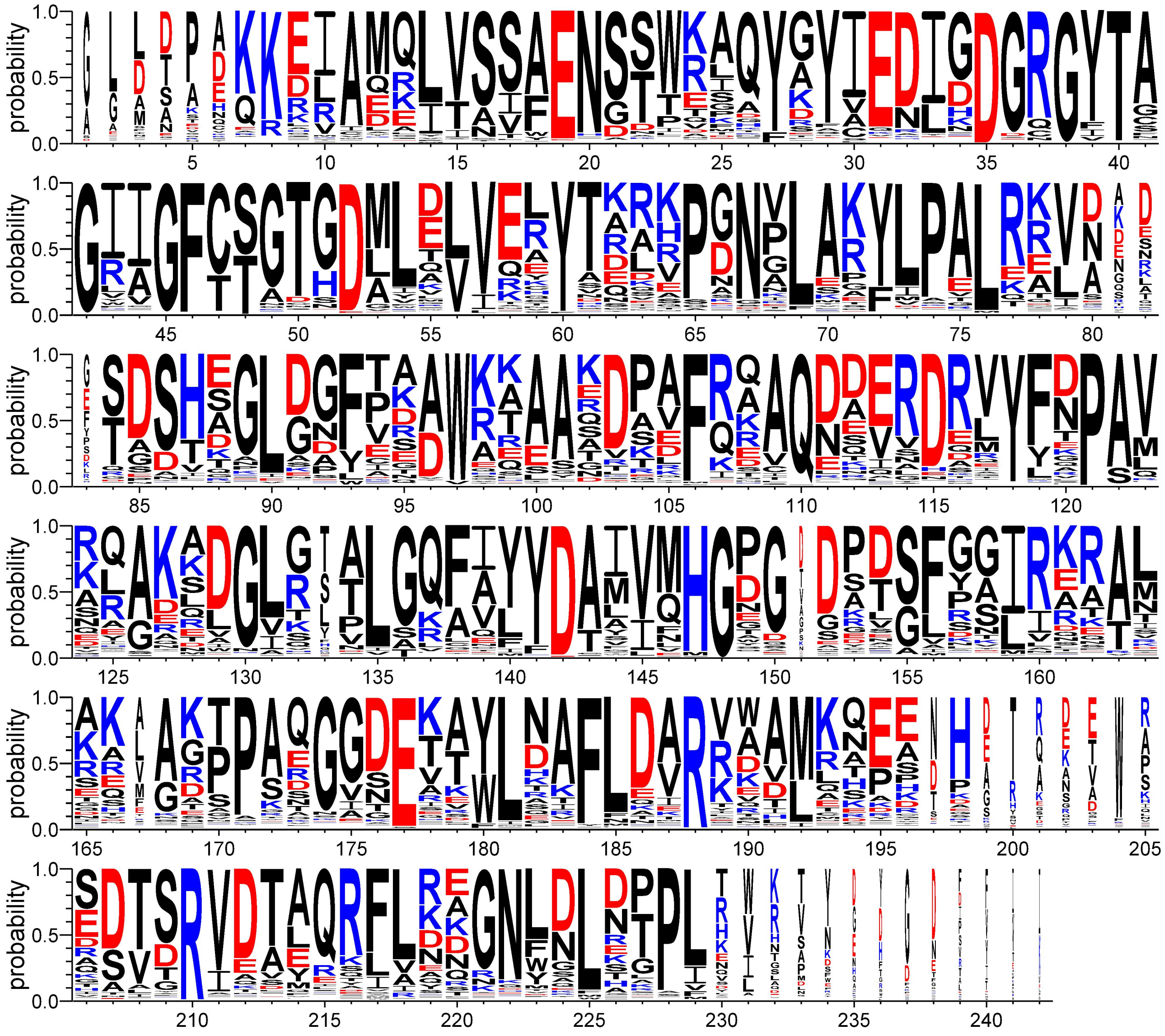

Figure S5. Consensus sequence analysis of CsnMY002 with 545 homologs.

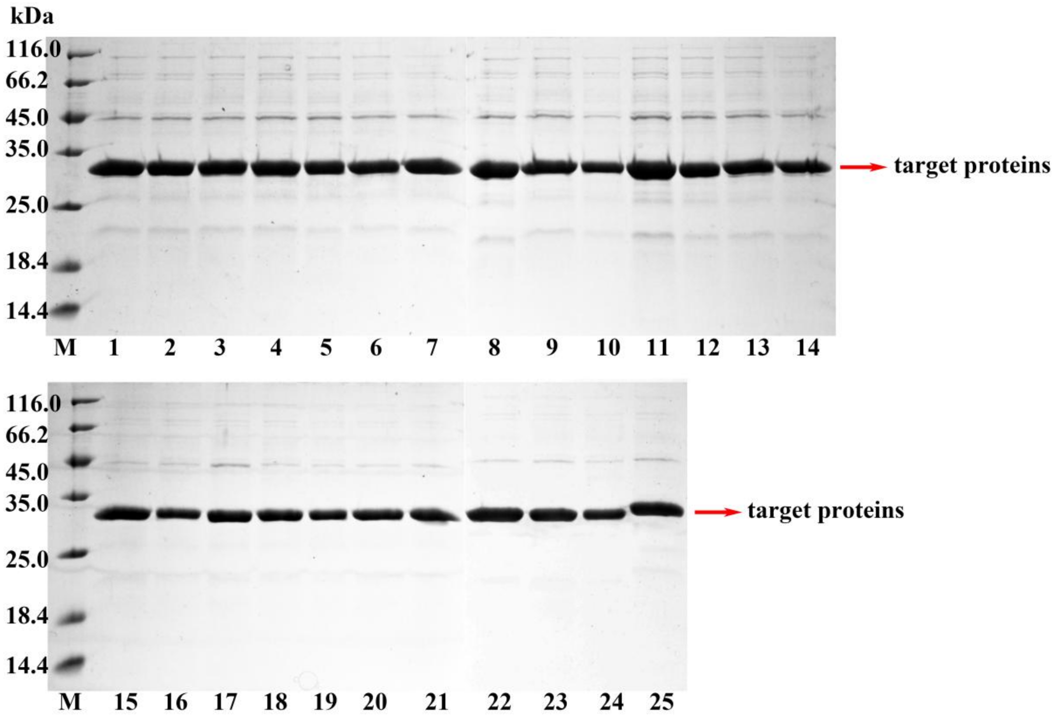

Figure S6. SDS-PAGE analysis of wild-type and mutants. Lanes 1–25 (in numerical order): wild-type, R32D, C41A, A49G, K70A, N89G, L100A, S126A, A136G, I175G, K183A, R215Q, S216R, A218L, N221G, E83Y, S84A, P195A, A196H, N197K, N197Y, H198L, H198W, D199L and D199R. M: Size markers.

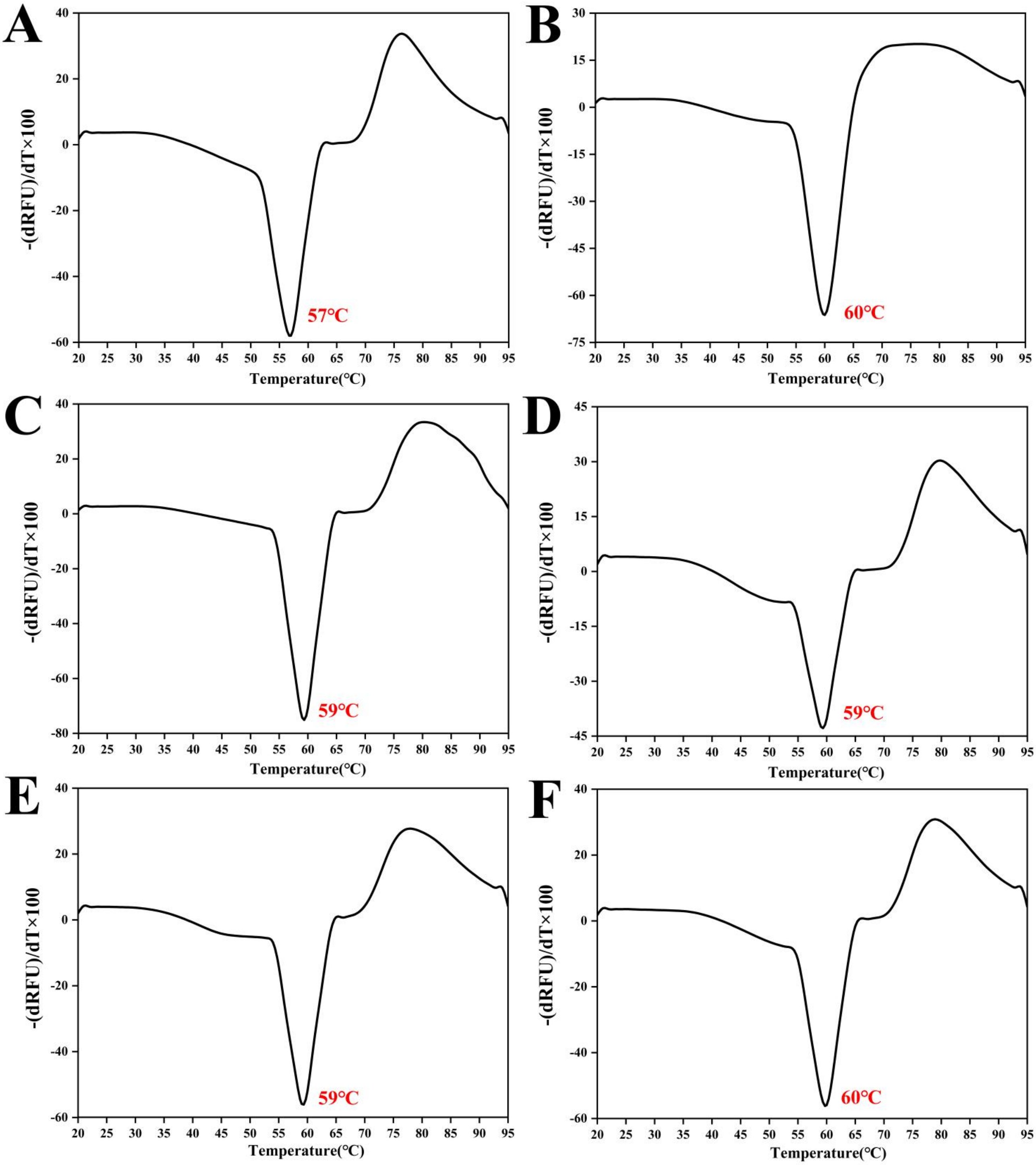

Figure S7. Measurement of the melting temperature (T_m) of wild-type and combination mutants. (A)Wild-type. (B) Mutant S84A/D199R. (C) Mutant K70A/N221G. (D) Mutant A49G/K70A/N221G. (E) Mutant A49G/K70A/N89G/N221G. (F) Mut6.

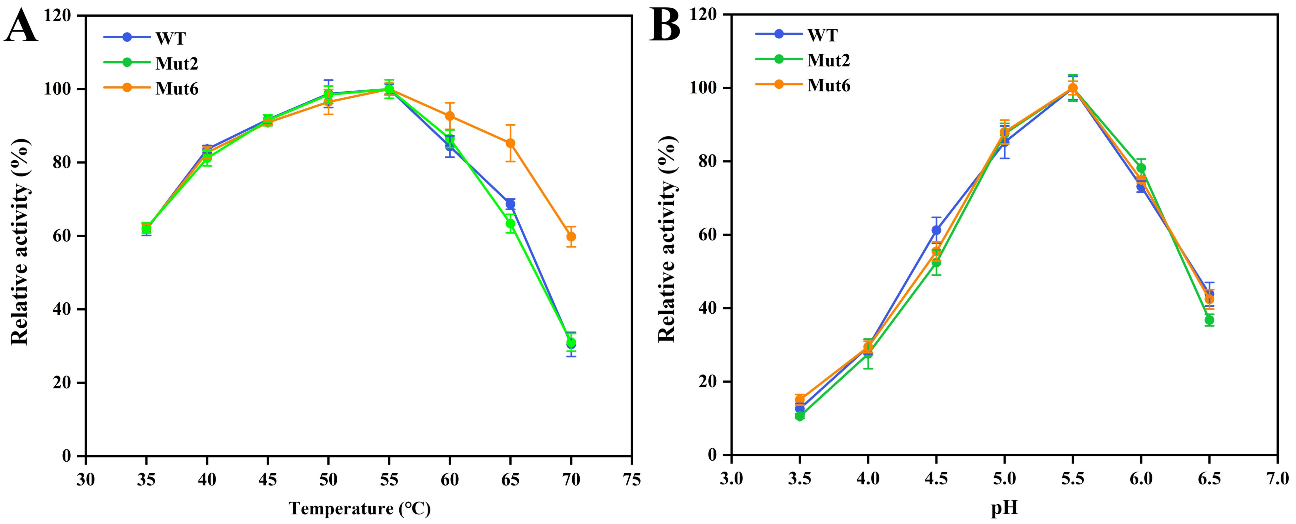

Figure S8. Measurement of optimum reaction conditions of wild-type (WT) and combination mutants. (A) The relative activity of the wild-type and the mutants at different temperatures. (B) The relative activity of the wild-type and the mutants at different pH values.

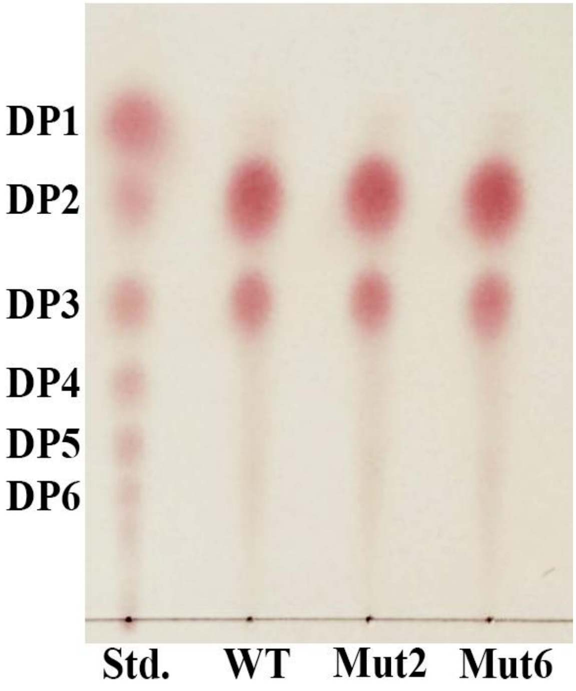

Figure S9. TLC analysis of hydrolysis products of wild-type (WT) and mutants.

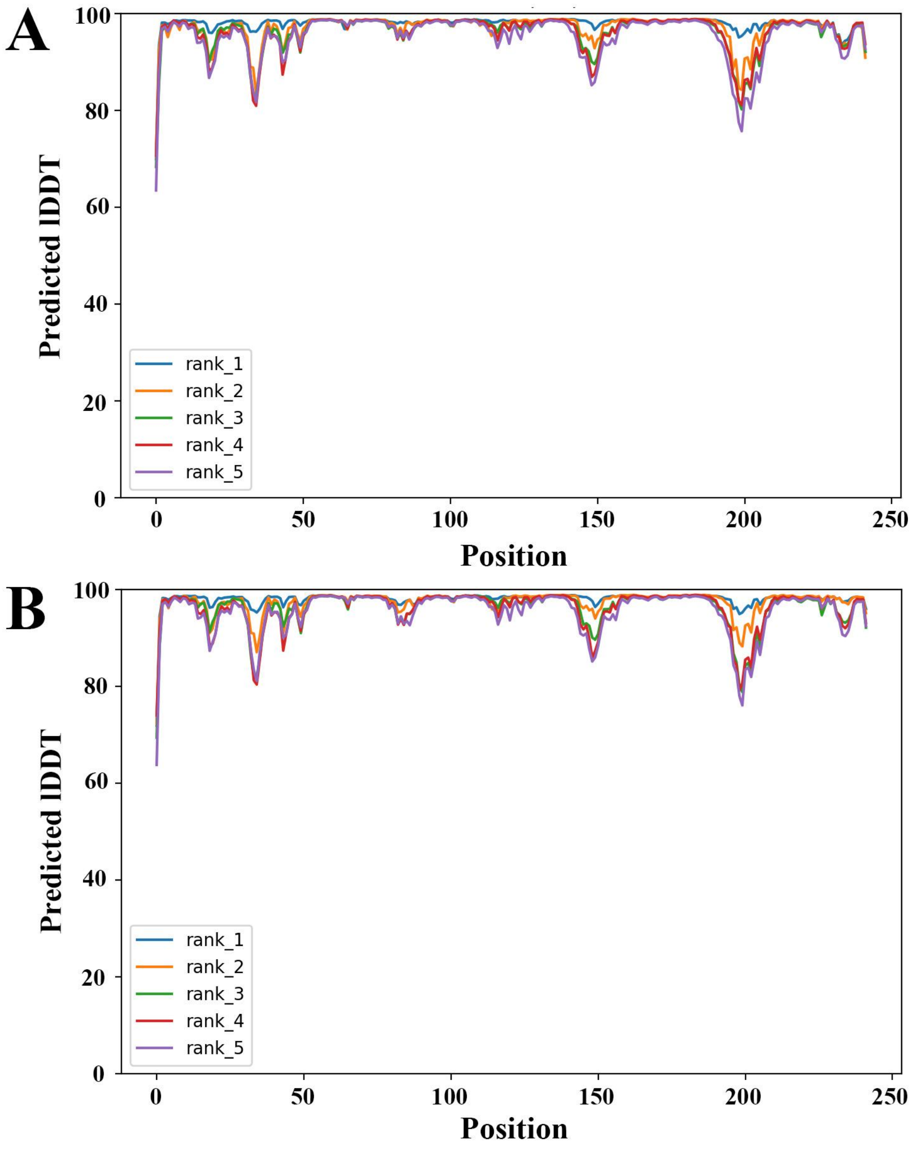

Figure S10. Quality assessment of the predicted models for Mut2 (A) and Mut6 (B). The ranks 1-5 represent the five prediction models generated by AlphaFold2 with high to low confidence levels.

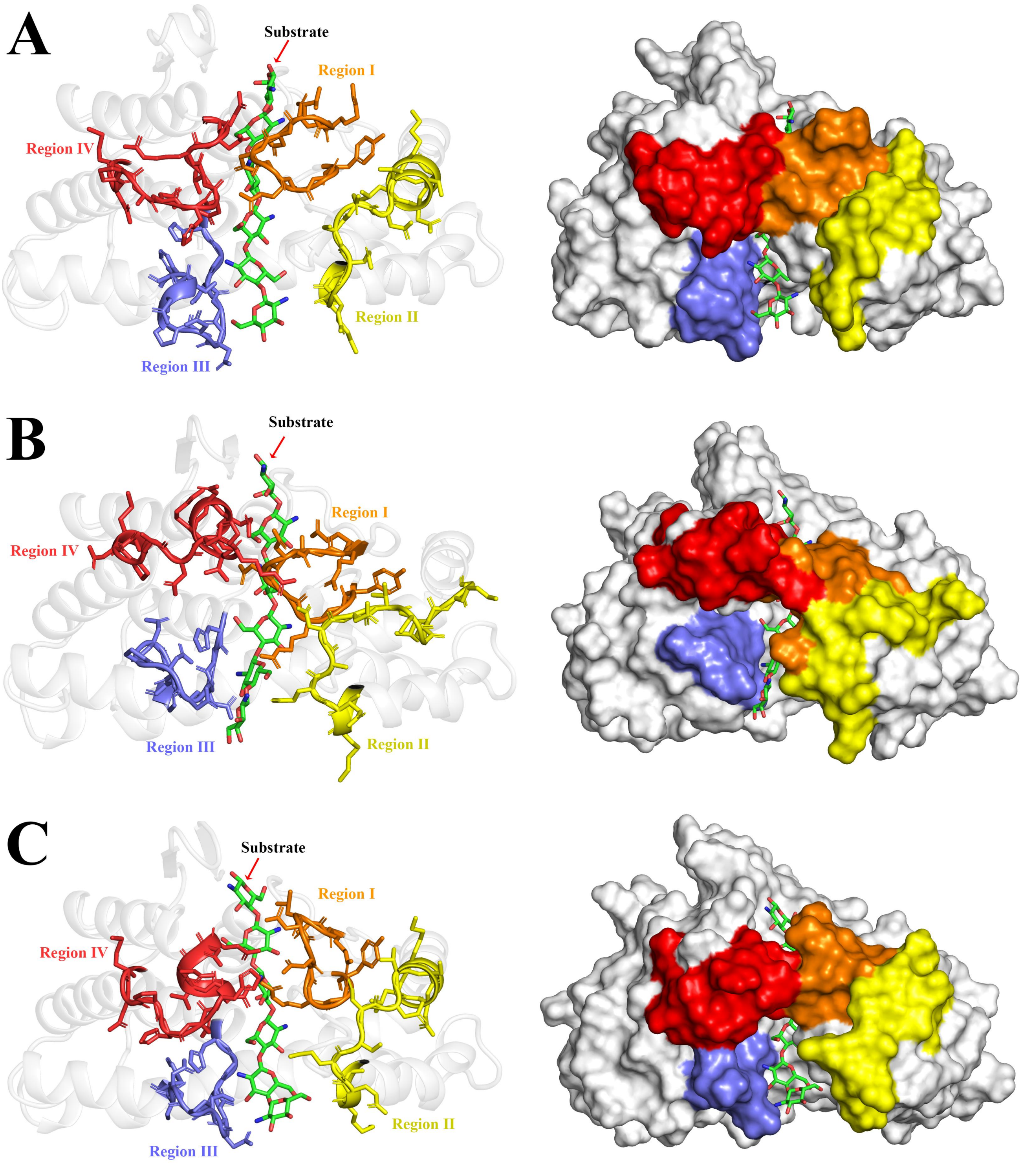

Figure S11. Analysis of dynamic regions of wild-type and mutant at 368K. (A) The dynamic regions of wild-type.(B) The dynamic regions of Mut6. (A) The dynamic regions of Mut2. The regions I, II, III and IV are shown in orange, yellow, blue and red, respectively. The substrates are represented by green sticks.
